# The effect of temperature on apoptosis and adipogenesis on skeletal muscle satellite cells derived from different muscle types

**DOI:** 10.14814/phy2.12539

**Published:** 2015-09-04

**Authors:** Rachel L Harding, Daniel L Clark, Orna Halevy, Cynthia S Coy, Shlomo Yahav, Sandra G Velleman

**Affiliations:** 1 Ohio Agricultural Research and Development Center, The Ohio State UniversityWooster, Ohio; 2 Department of Animal Sciences, The Hebrew University of JerusalemRehovot, Israel; 3 Institute of Animal Sciences, Agricultural Research Organization, The Volcani CenterBet Dagan, Israel

**Keywords:** Adipogenesis, apoptosis, fiber type, muscle, myogenic satellite cell, temperature

## Abstract

Satellite cells are multipotential stem cells that mediate postnatal muscle growth and respond differently to temperature based upon aerobic versus anaerobic fiber-type origin. The objective of this study was to determine how temperatures below and above the control, 38°C, affect the fate of satellite cells isolated from the anaerobic pectoralis major (p. major) or mixed fiber biceps femoris (b. femoris). At all sampling times, p. major and b. femoris cells accumulated less lipid when incubated at low temperatures and more lipid at elevated temperatures compared to the control. Satellite cells isolated from the p. major were more sensitive to temperature as they accumulated more lipid at elevated temperatures compared to b. femoris cells. Expression of adipogenic genes, CCAAT/enhancer-binding protein *β* (C/EBP*β*) and proliferator-activated receptor gamma (PPAR*γ*) were different within satellite cells isolated from the p. major or b. femoris. At 72 h of proliferation, C/EBP*β* expression increased with increasing temperature in both cell types, while PPAR*γ* expression decreased with increasing temperature in p. major satellite cells. At 48 h of differentiation, both C/EBP*β* and PPAR*γ* expression increased in the p. major and decreased in the b. femoris, with increasing temperature. Flow cytometry measured apoptotic markers for early apoptosis (Annexin-V-PE) or late apoptosis (7-AAD), showing less than 1% of apoptotic satellite cells throughout all experimental conditions, therefore, apoptosis was considered biologically not significant. The results support that anaerobic p. major satellite cells are more predisposed to adipogenic conversion than aerobic b. femoris cells when thermally challenged.

## Introduction

There are four types of muscle fibers: type I slow-twitch oxidative aerobic, type IIB fast-twitch glycolytic anaerobic, and intermediate types IIA and IIX (Quiroz-Rothe and Rivero 2004; Lee et al. 2010). The proportion of fiber types present in a mature muscle are based on the physiological requirements of the muscle (Quiroz-Rothe and Rivero 2004; Westerblad et al. 2010). Slow-twitch fibers comprise aerobic muscle that generates energy through oxidative breakdown of glucose within cellular mitochondria (Lee et al. 2010; Westerblad et al. 2010; Velleman and McFarland 2014). Aerobic muscles are fatigue resistant, have a greater blood supply, and are frequently involved in low energy tasks such as maintaining posture and other endurance tasks (Westerblad et al. 2010; Velleman and McFarland 2014). Alternately, fast-twitch anaerobic muscles provide short large bursts of energy through glycolysis of intramuscular glucose and are primarily utilized during rapid movements (Westerblad et al. 2010). Exercise, temperature, or disease can cause the fibers to transition from one fiber type to another (Gollnick et al. 1972; Liu et al. 2005; McClelland et al. 2006; Yamaguchi et al. 2010; Venhoff et al. 2012).

The extraordinary plasticity of skeletal muscle is largely due to the activity of adult myogenic stem cells, known as satellite cells (Hawke and Garry 2001). All postnatal muscle growth is mediated by satellite cells (Smith 1963; Hawke and Garry 2001) which were first identified by Mauro (1961) and are located between the basement membrane and sarcolemma of myofibers. Postnatal muscle growth or regeneration following injury occurs through a process called hypertrophy, when satellite cells proliferate, align, and fuse with myofibers thereby donating their nuclei. Hypertrophy increases protein synthesis of the myofiber resulting in myofiber enlargement (Moss and LeBlond 1971; Campion 1984).

Myogenic satellite cells are a highly heterogeneous population of stem cells. Satellite cell heterogeneity exists between satellite cells taken from different fiber types (Feldman and Stockdale 1991; Lagord et al. 1998; Huang et al. 2006; Manzano et al. 2011) in terms of variable rates of proliferation (McFarland et al. 1997; Collins et al. 2005; Manzano et al. 2011), differential expression of myogenic regulatory factors (Lagord et al. 1998; Manzano et al. 2011), and varying myogenic potentials (Lagord et al. 1998; Yada et al. 2006; Powell et al. 2014a). Even satellite cells from the same fiber are heterogeneous and vary in proliferation rate (McFarland et al. 1995; Rossi et al. 2010), gene expression (Zammit et al. 2004), and response to signaling molecules or growth factors (McFarland et al. 1995, 2003; Yun et al. 1997; Zeng et al. 2002).

As stem cells, satellite cells are also able to transdifferentiate to other cellular lineages including osteoblasts and adipocytes (Asakura et al. 2001; Shefer et al. 2004). Rossi et al. (2010) discovered a small subpopulation of satellite cells that possess the ability to spontaneously transdifferentiate to an adipogenic lineage. The differentiation of adipocytes is largely controlled by the transcription factor peroxisome proliferator-activated receptor gamma (PPAR*γ*) (Rosen et al. 1999; Rosen and MacDougald 2006; Vettor et al. 2009). The expression of PPAR*γ* is required for adipogenic differentiation and is considered the master regulator of adipogenesis. However, adipogenesis is not controlled by PPAR*γ* alone. For example, the CCAAT/enhancer-binding protein (C/EBP) family of proteins promote adipogenic differentiation as well as the expression (Rosen and MacDougald 2006) and activity (Hu et al. 1995) of PPAR*γ*. Current evidence suggests that CCAAT/enhancer-binding protein *β* (C/EBP*β*) and CCAAT/enhancer-binding protein *δ* (C/EBP*δ*) induce C/EBP*α* expression which directly promotes several adipogenic genes, including PPAR*γ* (Rosen and MacDougald 2006). Given these roles, PPAR*γ* and the C/EBP family of genes are frequently used as markers of adipogenesis.

Environmental factors and disease states have also been shown to alter skeletal muscle apoptosis. Although some apoptosis is normal during development (Sandri and Carraro 1999), apoptosis appears to be involved in muscle degeneration in conditions such as Duchenne muscular dystrophy (Tidball et al. 1995; Sandri and Carraro 1999; Sandri et al. 2001). Additionally, apoptosis is also at least partially responsible for muscle loss caused by atrophy due to lack of use or injury (Allen et al. 1997; Adhihetty et al. 2007) and is elevated following muscle injury and during repair in older animals (Siu et al. 2005; Marzetti et al. 2008). Thermal stress has been shown to decrease skeletal muscle growth by reducing hypertrophy (Friar and Locke 2007), and increase proteolysis of chick myotubes in culture (Nakashima et al. 2004). The satellite cell response to thermal stress in terms of apoptosis is not known, however, thermal stress may activate apoptotic pathways similar to that which was observed by Pophal et al. (2003) and Nierobisz et al. (2009) during nutritional deprivation in poultry.

The objective of the current study was to determine how temperatures both below and above the normal in vitro temperature of 38°C affects the behavior of satellite cells isolated from chicken p. major and b. femoris muscles, in regard to apoptosis and adipogenic potential of myogenic satellite cells. Data generated from the current study will provide an initial basis for understanding the effects of fiber type and temperature on satellite cell function in muscle development, growth, and conversion to an adipogenic lineage.

## Materials and Methods

### Isolation of broiler pectoralis major and biceps femoris satellite cells

Satellite cells were previously isolated from the p. major muscle or b. femoris muscle of 5-week-old female Cornish Rock broiler chickens and pooled (*Gallus domesticus*). Single satellite cells were isolated to create a clonal population using a Quixell cell manipulator robotic system (Stoelting Co., Wood Dale, IL). Clonal populations were expanded, and stored in liquid nitrogen until use (McFarland et al. 1997). This isolation produced a homogenous satellite cell population free of fibroblast and other nonmyogenic cell types.

### Cell culture

Broiler p. major and b. femoris satellite cells were plated simultaneously in 24-well 0.1% porcine gelatin (Sigma-Aldrich, St. Louis, MO)-coated cell culture plates (Gemini BioProducts, West Sacramento, CA) at 12 000 cells per well for each experimental comparison. Plating was performed with medium consisting of Dulbecco's modified Eagle's Medium (DMEM; Sigma-Aldrich) with 10% chicken serum, 5% horse serum, 1% antibiotic/antimycotic, and 0.1% gentamicin (Gemini BioProducts). Plates were incubated in a 95% air/5% CO_2_ incubator (Thermo Fisher Scientific, Pittsburgh, PA) at 38°C. Following 24 h for attachment, the medium was changed to feeding medium containing McCoy's 5A (Sigma-Aldrich) with 10% chicken serum, 5% horse serum, 1% antibiotic/antimycotic, and 0.1% gentamicin (Gemini BioProducts). Plates for the experimental temperatures below or above control (33, 35, 37, 39, 41, or 43°C) were moved to a separate incubator (95% air/5% CO_2_) (Thermo Fisher Scientific) at the desired experimental temperature following attachment of the satellite cells, while control cultures remained at 38°C. Once the cultures reached 72 h following attachment (approximately 60–65% confluency), differentiation was induced with a low-serum DMEM medium containing 3% horse serum, 1% antibiotic/antimycotic, 0.1% gentamicin (Gemini BioProducts), 0.1% porcine gelatin, and 1 mg/mL bovine serum albumin (Sigma-Aldrich). Medium was changed every 24 through 72 h of proliferation and 72 h of differentiation. Digital photomicrographs of the satellite cell cultures were taken every 24 h during proliferation and differentiation using an Olympus IX70 fluorescent microscope (Olympus America, Melville, NY) and QImaging digital camera (QImaging, Burnaby, BC, Canada) equipped with CellSens Imaging software (Olympus America).

### Quantitation and imaging of lipid accumulation

Lipid accumulation in satellite cell cultures was measured by AdipoRed (Lonza Inc., Walkersville, MD) quantitation at both the control (38°C) and experimental temperatures (33, 35, 37, 39, 41, or 43°C). Satellite cells were cultured as described with lipid accumulation measured at 72 h of proliferation, and 48 and 72 h of differentiation. AdipoRed quantitation was performed according to the manufacturer's protocol. In brief, plates were removed from the cell culture incubator, the media was removed, and wells were rinsed with 1 mL phosphate-buffered saline (PBS: 137 mmol/L NaCl, 2.68 mmol/L KCl, 1.47 mmol/L KH_2_PO_4_, and 7.81 mmol/L Na_2_HPO_4_, pH 7.08). An additional 1 mL of PBS was added to each cell well and two blank wells as controls for AdipoRed incorporation. To each cell culture well and one control well, 30 *μ*L AdipoRed was added and mixed by pipette. Plates were incubated with the AdipoRed for 15 min at room temperature and read on a Fluorskan Ascent FL scanner (Thermo Fisher Scientific) with an excitation of 485 nm and emission of 538 nm. Experiments were plated four times with four replicate wells per cell type, experiment, and sampling time.

Lipid containing cells in the satellite cell cultures were detected with Oil Red O (Sigma-Aldrich) staining at 72 h of proliferation, and 48 h and 72 h of differentiation. Plates were removed from the incubator, media removed and fixed in 500 *μ*L of 10% formalin (Electron Microscopy Sciences, Hatfield, PA) for 5 min. The initial 10% formalin was removed and replaced with an equal volume of fresh 10% formalin for at least 1 h at room temperature. Cells were then washed with 60% isopropanol then allowed to dry completely. Once dry, 200 *μ*L 0.5% Oil Red O was added to each well for 10 min. The plates were then washed under gentle running water for about 5 min until all the non-specific Oil Red O was removed. All plates were allowed to dry before 250 *μ*L of 2 *μ*g/mL of 4′,6-diamidino-2-phenylindole (DAPI; Biotium, Hayward, CA) was added to each well for 20 min to stain cellular nuclei. The DAPI was removed and each well was washed with PBS. Labeled cells were stored in PBS at 4°C until imaging with an Olympus IX70 fluorescent microscope and a QImaging digital camera using CellSens software. Two separate experiments were plated with four replicate wells per experiment per sampling time.

### Measurement of apoptotic cells

For measurement of apoptotic cells, chicken broiler p. major and b. femoris muscle satellite cells were cultured as described above in 24-well culture plates, with the temperature experimental plates being transferred to the variable temperature cell culture incubator following 24 h of attachment. Cell cultures were collected at 48 h of proliferation, and 24 or 48 h of differentiation for flow cytometry analysis of apoptosis and cell size. Media from each well was transferred to a separate 1.5 mL tube. Cells were then treated with 100 *μ*L of 0.25% trypsin/EDTA in PBS (Invitrogen) for 3 min at room temperature. Cells from each well were then transferred to corresponding media tubes and centrifuged for 10 min at 750 *g*. The supernatant was then discarded and cells were resuspended in 1.2 mL fresh feeding media at a concentration between 2 × 10^5^ and 1 × 10^6^ cells/mL.

**Figure 1:**
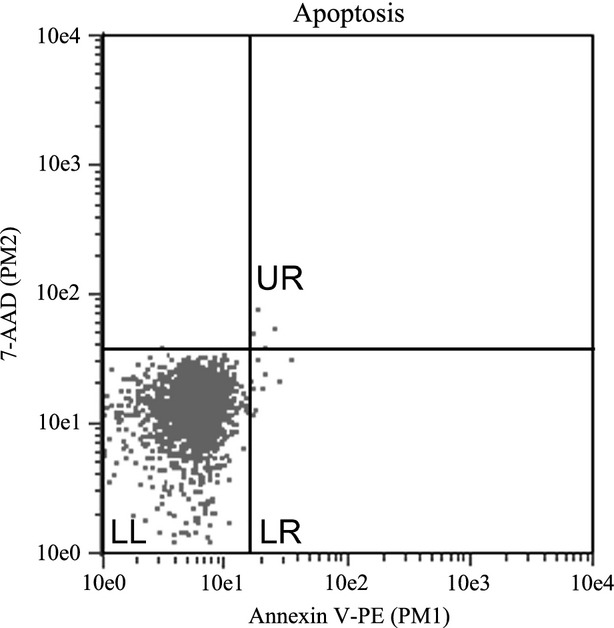
Example of apoptosis gating of satellite cells. Gates were placed at apparent breaks between cell populations and remained in the same position for both the pectoralis major and biceps femoris satellite cells at all temperatures for sampling time of each experiment. Cells in the lower left quadrant (LL) were negative for both early apoptosis marker Annexin-V conjugated to phycoerythrin (Annexin-V-PE) and late apoptosis marker 7-amino-actinomycin D (7-AAD), therefore are not undergoing apoptosis. Cells in the lower right quadrant (LR) were Annexin-V-PE positive, 7-AAD negative, marking them as early apoptotic cells. Cells in the upper right (UR) quadrant were positive for Annexin-V-PE and 7-AAD indicating late apoptosis.

Apoptosis was detected by flow cytometry using the Guava Nexin procedure (EMD Millipore Guava Technologies, Inc., Billerica, MA) according to manufacturer's protocols. When performing the Guava Nexin (EMD Millipore Guava Technologies) assay, the Guava Nexin reagent contains indicators of both early and late apoptosis. Early apoptosis is detected with Annexin-V-conjugated to phycoerythrin (Annexin-V-PE) which binds phosphatidylserine present on the external surface of cells that are in early stages of apoptosis. Late apoptosis is detected by the 7-amino-actinomycin D (7-AAD) dye that is only taken up by structurally compromised cells in later stages of apoptosis. Due to these labeling conditions, early apoptotic cells were Annexin-V-PE positive and 7-AAD negative (Annexin-V-PE^+^/7-AAD^−^), while late apoptotic cells were Annexin-V-PE positive and 7-AAD positive (Annexin-V-PE^+^/7-AAD^+^). For each sample, 100 *μ*L of cells resuspended at a concentration between 2 × 10^5^ and 1 × 10^6^ cells/mL were combined with 100 *μ*L of Guava Nexin reagent in a clear flat bottomed 96-well plate (Greiner Bio-one, Monroe, NC). The cells were then incubated in the dark for 20 min. Plates were read on the Guava EasyCyte Plus flow cytometry system (EMD Millipore Guava Technologies) according to the manufacturer's Guava Nexin apoptosis instructions. Samples were gated with X and Y intercepts between 10 (10e1) and 20 (10e1.3) on a log-fold scale at apparent breaks in cell populations as illustrated in Figure 1. Once gated, cells within the lower left quadrant were not labeled with either marker, while cells within the lower right quadrant were Annexin-V-PE^+^/7-AAD^−^, and those in the upper right quadrant were Annexin-V-PE^+^/7-AAD^+^. Very few cells were in the upper left quadrant and were not positive for the early apoptotic marker Annexin-V-PE, so were not considered. Four experiments were independently plated with four replicate wells of each cell type per experiment for apoptosis.

### Real-time quantitative PCR analysis of gene expression

Both p. major and b. femoris satellite cells were cultured for gene expression analysis as described above at the control (38°C) temperature and variable experimental temperatures (33, 35, 37, 39, 41, or 43°C) and collected at 72 h of proliferation and 48 h of differentiation. Plates were removed from the cell culture incubator, media removed, culture wells rinsed with PBS, air dried, and stored at −70°C until analysis. Experiments were plated with a total of six-well replicates per treatment and sampling time. All six replicates from each treatment and sampling time were pooled for RNA extraction. Total RNA was isolated from cell culture plates using RNAzol RT (Molecular Research Center Inc., Cincinnati, OH) according to the manufacturer's protocol. Reverse transcription to produce cDNA was completed using Moloney murine leukemia virus (MMLV) reverse transcriptase reagents (Promega, Madison, WI). Briefly, 1 *μ*g of total RNA was combined with 1 *μ*L 50 mmol/L Oligo dT (Operon, Huntsville, AL) and nuclease-free water to a volume of 13.5 *μ*L and incubated at 70°C for 5 min, then placed on ice. A mixture of reaction reagents containing 5 *μ*L of 5 × MMLV buffer, 1 *μ*L of 10 mmol/L deoxynucleotide triphosphate mix, 0.25 *μ*L of 40 U/*μ*L RNAsin, 1 *μ*L of 200 U/*μ*L MMLV reverse transcriptase, and nuclease-free water up to 11.5 *μ*L per reaction were added to each tube. Samples were then incubated at 55°C for 60 min and 90°C for 10 min. Following incubation, 25 *μ*L of nuclease-free water was added to each reaction. Real-time quantitative PCR (RT-qPCR) was performed using DyNAmo Hot Start SYBR green qPCR master mix (Finnzymes, Ipswich, MA) according to manufacturer's instructions for analysis of glyceraldehyde-3-phosphate dehydrogenase (GAPDH), PPAR*γ*, C/EBP*α*, and C/EBP*β* expression. Primer sequences and GenBank accession numbers are listed in Table 1. Primer specificities were confirmed by DNA sequencing of gel-purified PCR products (Molecular and Cellular Imaging Center, The Ohio Agricultural Research and Development Center, Wooster; Powell et al. 2014a, Velleman and McFarland 2014). In brief, 2 *μ*L of cDNA was combined with a reaction mix containing 10 *μ*L 2 × DyNAmo HS SYBR green master mix, 1 *μ*L of primer mix containing 10 *μ*mol/L each forward and reverse primers, and 7 *μ*L of nuclease-free water. Reactions were run on a DNA Engine Opticon 2 real-time machine (BioRad, Hercules, CA) with the following cycling conditions: 95°C for 15 min, 34 cycles of 94°C for 30 sec, 55°C for 30 sec, and 72°C for 30 sec, followed by a final extension at 72°C for 5 min. Amplification specificity was confirmed by resolving randomly selected samples from all RT-qPCR reactions on a 1% agarose gel. Six serial dilutions of purified PCR products were used to produce standard curves for each gene. Serial dilutions were assigned arbitrary concentrations between 1 and 1 × 10^6^. The arbitrary values of samples were calculated based on the relation of curve Ct values to assigned standard curve concentrations. Data were then normalized to the average GAPDH expression of pooled samples from all 38°C treatments by dividing the arbitrary molar concentration of the samples by the arbitrary GAPDH molar value for each cell type at each sampling time as GAPDH expression was affected by temperature. Two experiments were plated with three technical replicate RT-qPCR reactions run per sample for each gene per experiment. Data from a single experiment were selected as a representative of both experiments analyzed.

**Table 1:** Primer sequences for genes analyzed by real-time quantitative PCR

Primer	Sequence	Coding region	Product size	GenBank number
GAPDH*	5′-GAG GGT AGT GAA GGC TGC TG-3′ (forward)	504–523	200 bp**	U94327.1
	5′-CCA CAA CAC GGT TGC TGT AT-3′ (reverse)	684–703		
C/EBP*α*†	5′-CAG TGG ACA AGA ACA GCA ACG A-3′ (forward)	728–749	227 bp	NM_001031459.1
	5′-CCT TCA CCA GCG AGC TTT CG-3′ (reverse)	936–955		
C/EBP*β*‡	5′-TCC TAC CTG GGC TAC CAG TC-3′ (forward)	791–810	169 bp	NM_205253.2
	5′-CGC ACT TCT TGG GCT TGT TC-3′ (reverse)	940–959		
PPAR*γ*§	5′-CCA CTG CAG GAA CAG AAC AA-3′ (forward)	805–824	249 bp	NM_001001460.1
	5′-CTC CCG TGT CAT GGA TCC TT-3′ (reverse)	1035–1054		

* Glyceraldehyde-3-phosphate dehydrogenase

† CCAAT/enhancer-binding protein alpha

‡ CCAAT/enhancer-binding protein beta

§ Peroxisome proliferator-activated receptor gamma

** base pairs of DNA

### Statistical analysis

Statistical analysis of GuavaNexin apoptosis data were performed using the PROC GLIMMIX procedure of Statistical Analysis System (SAS; SAS Institute, 2011). Four independent culture experiments were plated for each experimental temperature. For each experiment, both satellite cell types were incubated at 38°C and an experimental temperature. Satellite cells of each cell type were plated in four wells for each temperature, which were treated at four replicates within each experiment. The percentage of late and early apoptotic cells were analyzed separately. To reduce the number of comparisons, two statistical analyses were performed. The first analysis was a one-way analysis of variance (ANOVA) completed within each temperature and cell type combination to determine differences across time; therefore, the model included the main effect of time and was blocked by experiment. The least square means (lsmeans) statement was used to determine the means and standard error of the mean. The pdiff option was used to separate the interaction means. The second analysis was completed to determine the effects of cell type and incubation temperature within time; therefore the treatments were arranged in a 2 × 2 factorial design within each sampling time. The model included the main effects of incubation temperature, cell type, their interaction, and the random effect of the block (experiment). The lsmeans statement was used to determine the means and standard error of the mean. The pdiff option was used to separate the interaction means. Differences were considered significant at *P *<* *0.05.

For gene expression, two experiments were plated with three technical replicate RT-qPCR reactions run per sample for each gene per experiment. Statistical analysis was performed on all experiments and data from a single experiment were selected as representative of both experiments analyzed. For the AdipoRed assay, four independent culture experiments were plated for each experimental temperature. For each experiment, both satellite cell types were incubated at 38°C and an experimental temperature. Satellite cells of each cell type were plated in four wells for each temperature, which were treated at four replicates within each experiment. Both datasets were regressed across incubation temperature within each sampling time in the MIXED procedure of SAS. Appropriate linear contrast statements were used to obtain the linear equation for each cell type across the experimental temperatures. An additional contrast statement was used to test for sensitivity differences between the linear responses across temperature and between the cell types. The lsmeans statement was used to determine the means and standard error of the mean. The model included the main effects of incubation temperature, cell type, their interaction, and the random effect of block (experiment). Simultaneously, the data were sliced by temperature to signify differences between cell types within each experimental temperature. An additional analysis was completed on AdipoRed data for the extreme temperatures (33°C and 43°C) compared to the respective control temperature to understand how lipid accumulation changes over time. A one-way ANOVA completed within each temperature and cell type combination was used to determine differences across time; therefore, the model included the main effect of time and was blocked by experiment. The lsmeans statement was used to determine the means and standard error of the mean. The pdiff option was used to the separate interaction means. All differences were considered significant at *P < *0.05.

## Results

### Effect of temperature on lipid accumulation

**Figure 2:**
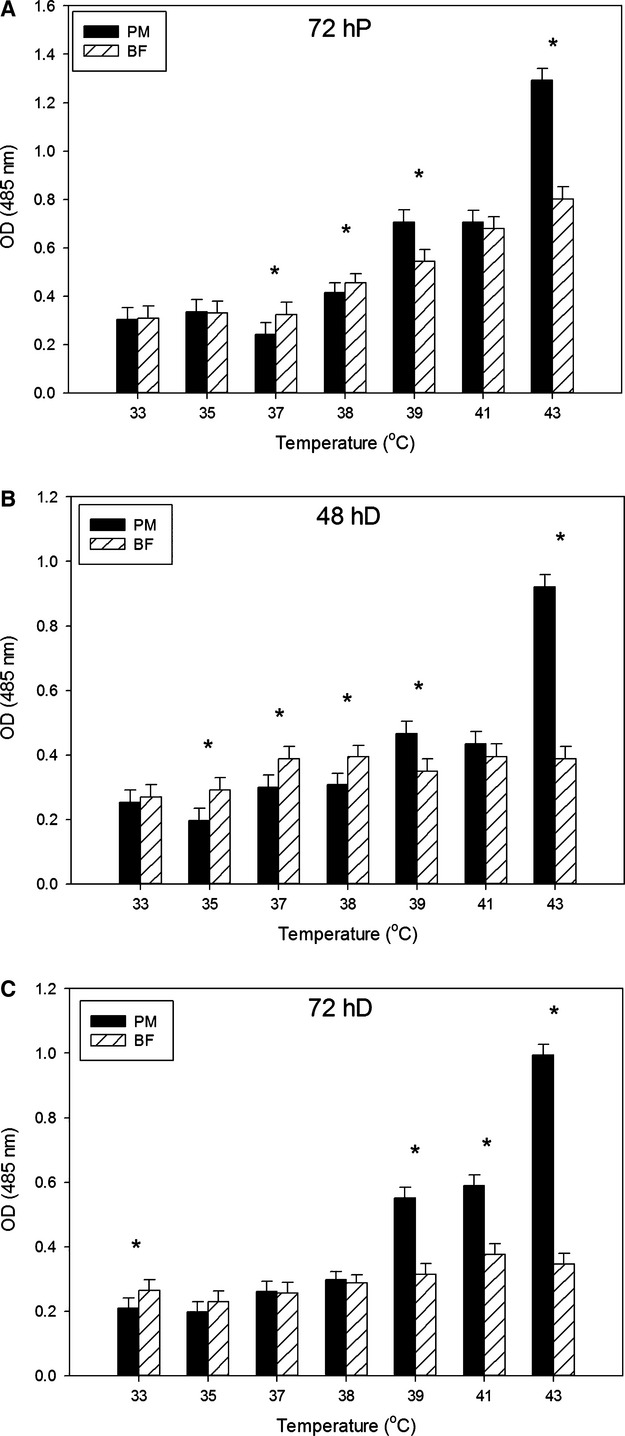
Analysis of the effect of temperature on lipid accumulation in pectoralis major and biceps femoris satellite cells during proliferation and differentiation. AdipoRed fluorescence as a measure of lipid accumulation in satellite cells incubated between 33°C and 43°C analyzed at 72 h of proliferation (A), 48 h of differentiation (B), and 72 h of differentiation (C). Bars represent standard error of the mean. PM, pectoralis major; BF, biceps femoris. Data with *indicate significant difference between cell types (*P *≤ 0.05).

Lipid accumulation was measured at temperatures below the control of 38°C (33, 35, and 37°C) or above the control (39, 41, and 43°C) at 72 h of proliferation, 48 and 72 h of differentiation. At 72 h of proliferation, p. major satellite cells accumulated significantly less (*P *≤* *0.03) lipid than b. femoris cells at 37 and 38°C (Fig. 2A). The opposite was true at 39 and 43°C, as p. major satellite cells accumulated more lipid (*P *<* *0.001) than b. femoris satellite cells (Fig. 2A). Lipid accumulation in both p. major and b. femoris satellite cells at 72 h of proliferation linearly increased (*P *<* *0.001) as temperature increased (33–43°C), with p. major satellite cells (slope: 0.09) having a greater increase (*P *<* *0.001) than b. femoris cells (slope: 0.04) (Fig. 2A). At 48 h of differentiation, p. major satellite cells accumulated less lipid (*P *<* *0.001) at 35, 37, and 38°C compared to b. femoris cells (Fig. 2B). At 39 and 43°C p. major cells contained significantly more (*P *<* *0.001) lipid than b. femoris cells, but there was no difference (*P = *0.06) in lipid accumulation between muscles at 41°C. At 48 h of differentiation, linear regression indicated that lipid accumulation increased in p. major satellite cells linearly (slope: 0.05) with increasing temperature (*P *<* *0.001), but lipid accumulation within the b. femoris satellite cells remained constant (slope: 0.003; *P *=* *0.34) across temperature (Fig. 2B, C). At 72 h of differentiation, p. major cells contained less (*P *<* *0.001) lipid than b. femoris cells at 33°C, but there was no difference between muscle types at 35, 37, or 38°C (Fig. 2C, *P *≥* *0.24). At temperatures above the control (39, 41, and 43°C) p. major satellite cells accumulated more lipid than b. femoris satellite cells at 72 h of differentiation (Fig. 2C, *P *<* *0.001). At 72 h of differentiation accumulation of lipid linearly increased within both p. major (slope: 0.08) and b. femoris (slope: 0.01) satellite cells with increasing temperature (*P *<* *0.001), but p. major satellite cells had a greater (*P < *0.001) rate of lipid accumulation than b. femoris cells (Fig. 2C).

Satellite cells grown at the lowest temperature of 33°C accumulated less lipid at all sampling times compared to satellite cells grown at the control temperature of 38°C (*P *<* *0.01) in both p. major and b. femoris satellite cells (Table 2). The differences between 38°C cultures and 33°C cultures at each sampling time were less significant in p. major satellite cell cultures compared to b. femoris cells. Comparison of lipid accumulation in each cell type across time showed minimal differences in the amount of quantified lipid. In p. major satellite cells grown at 38°C, lipid accumulation was not affected (*P *=* *0.11) by time, whereas lipid accumulation within p. major satellite cells grown at 33°C increased (*P *<* *0.01) from 72 h of proliferation to 48 h of differentiation and then decreased (*P *<* *0.01) from 48 h of differentiation to 72 h of differentiation. A reduction (*P *<* *0.05) in accumulated lipid was observed in b. femoris cells at 72 h of differentiation in 38°C cultures. Increased (*P *<* *0.001) lipid accumulation was observed at 48 and 72 h of differentiation in b. femoris 33°C cultures.

**Table 2:** Effect of temperature on lipid accumulation at 38 and 33°C during proliferation (P) and differentiation (D)*

Time	PM 38°C	PM 33°C	BF 38°C	BF 33°C
72 hP	0.24 ± 0.03^b^,^x^	0.15 ± 0.03^c^,^y^	0.32 ± 0.03^a^,^x^	0.16 ± 0.03^c^,^y^
48 hD	0.24 ± 0.01^b^,^x^	0.19 ± 0.01^c^,^x^	0.34 ± 0.01^a^,^x^	0.21 ± 0.01^c^,^x^
72 hD	0.20 ± 0.01^b^,^x^	0.15 ± 0.01^c^,^y^	0.26 ± 0.01^a^,^y^	0.20 ± 0.01^b^,^x^

PM, pectoralis major; BF, biceps femoris.

a-c Means across cell type and temperature at each sampling time; values without common letters are different (*P *< 0.05).

x-y Means across sampling times for each cell type and temperature combination; values without common letters are different (*P *< 0.05).

* Lipid accumulation was quantified through mean fluorescence of AdipoRed labeling (±SEM).

Both p. major and b. femoris satellite cells incubated at 43°C accumulated more lipid (*P *<* *0.01) than 38°C cultures, except at 48 h of differentiation when b. femoris cultures at 38°C accumulated equivalent (*P *≥* *0.75) amounts of lipid compared to b. femoris satellite cells grown at 43°C (Table 3). Additionally, p. major satellite cells grown at 43°C accumulated more (*P *<* *0.001) lipid than b. femoris cells grown at 43°C. Examining these data across time, 38°C cultures of p. major satellite cells had decreased (*P *<* *0.004) lipid accumulation at 48 h differentiation, but a slight increase at 72 h differentiation making the lipid accumulation statistically equivalent (*P *>* *0.10) to both 72 h proliferation and 48 h differentiation. The b. femoris satellite cells at 38°C had decreased (*P *<* *0.002) lipid accumulation at 72 h differentiation compared to earlier sampling times. In contrast, both p. major and b. femoris cells at 43°C had decreased (*P *<* *0.001) lipid accumulation at both 48 and 72 h differentiation compared to 72 h proliferation.

**Table 3:** Effect of temperature on lipid accumulation at 38 and 43°C during proliferation (P) and differentiation (D)*

Time	PM 38°C	PM 43°C	BF 38°C	BF 43°C
72 hP	0.36 ± 0.06^c^,^x^	1.21 ± 0.06^a^,^x^	0.35 ± 0.06^c^,^x^	0.73 ± 0.06^b^,^x^
48 hD	0.29 ± 0.03^c^,^y^	0.88 ± 0.03^a^,^y^	0.34 ± 0.03^b^,^x^	0.21 ± 0.03^b^,^y^
72 hD	0.32 ± 0.04^bc^,^xy^	0.98 ± 0.04^a^,^y^	0.24 ± 0.04^c^,^y^	0.33 ± 0.04^b^,^y^

PM, pectoralis major; BF, biceps femoris.

a–c Means across cell type and temperature at each sampling time; values without common letters are different (*P *< 0.05).

x–y Means across sampling times for each cell type and temperature combination; values without common letters are different (*P *< 0.05).

* Lipid accumulation was quantified through mean fluorescence of AdipoRed labeling (±SEM).

**Figure 3:**
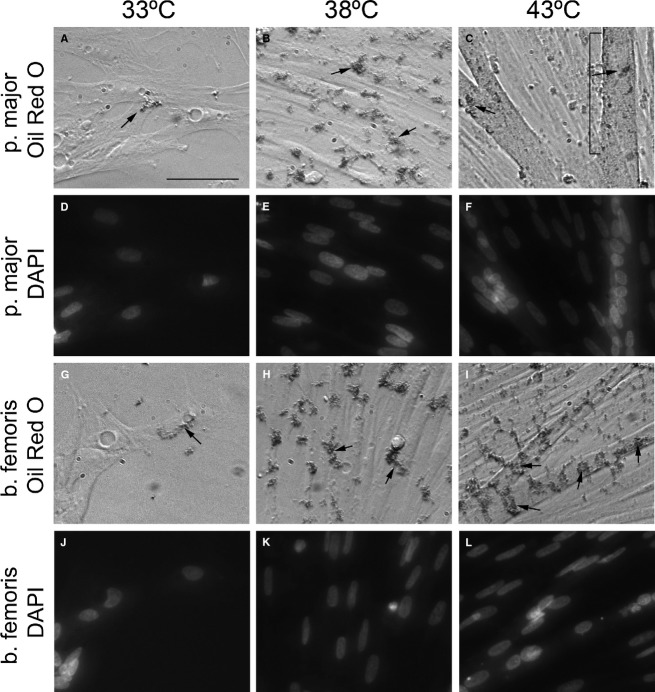
Accumulated lipid in pectoralis major (p. major) and biceps femoris (b. femoris) satellite cells at different temperatures. Lipid accumulation at 72 h differentiation labeled with Oil Red O in myogenic satellite cell cultures grown at 33°C (A, G), 38°C (B, H), and 43°C (C, I) from p. major (A–C) and b. femoris (G–I) muscle. Nuclei were labeled with 4′,6-diamidino-2-phenylindole (DAPI) in the myogenic satellite cells from p. major (D–F) and b. femoris (J–L) muscles at 33°C (D, J), 38°C (E, K), and 43°C (F, L). Scale bar = 50 *μ*m.

Pectoralis major and b. femoris satellite cells incubated at 33, 38, or 43°C were microscopically analyzed for lipid accumulation within cells with fat soluble Oil Red O staining at 72 h of differentiation (Fig. 3). Minimal lipid accumulation was observed in both p. major and b. femoris satellite cells and myotubes incubated at 33°C (Fig. 3A, G, arrows) compared to 38°C (Fig. 3B, H, arrows). For those cultures grown at 43°C, lipid staining was readily observable in both p. major (Fig. 3C, arrows) and b. femoris satellite cells (Fig. 3I, arrows), and more prevalent than in cultures grown at 38°C (Fig. 3B, H). In several cases, p. major and b. femoris myotubes incubated at 43°C contained both clusters of lipids (Fig. 3C, arrow) and more diffuse staining (Fig. 3C, brackets), making the lipid presence nearly uniform throughout the myotube. Satellite cells and myotubes in all experimental conditions had clearly labeled DAPI-positive nuclei (Fig. 3D–F, J–L), demonstrating lipid accumulation within the satellite cells and myotubes.

**Figure 4:**
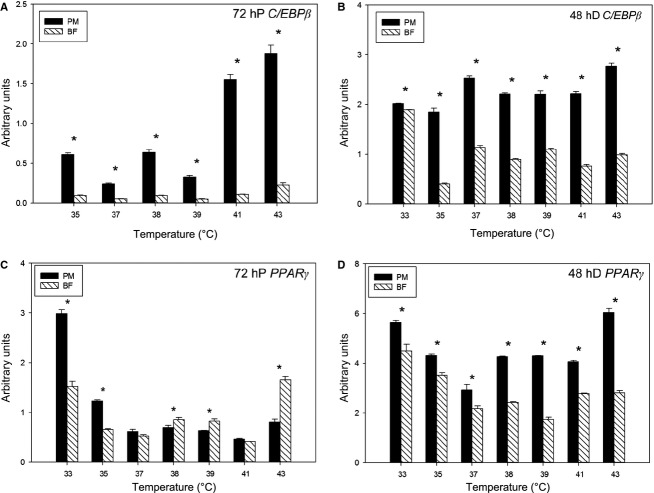
Expression of adipogenic genes CCAAT/enhancer-binding protein *β* and peroxisome proliferator-activated receptor gamma in pectoralis major and biceps femoris satellite cells at different temperatures during proliferation and differentiation. The expression of CCAAT/enhancer-binding protein *β* (C/EBP*β*) at (A) 72 h of proliferation and (B) 48 h of differentiation, and peroxisome proliferator-activated receptor gamma (PPAR*γ*) at (C) 72 h of proliferation, and (D) 48 h of differentiation by real-time quantitative PCR. Bars represent standard error of the mean. PM, pectoralis major; BF, biceps femoris. 72 hP, 72 h of proliferation; 48 h D, 48 h of differentiation. Data with *indicate significant difference between cell fiber types (*P *≤ 0.05) at individual temperatures.

Expression of C/EBP*β* was significantly higher (*P *<* *0.05) in p. major satellite cells compared to b. femoris cells at both 72 h of proliferation (Fig. 4A), and 48 h of differentiation (Fig. 4B) at all culture temperatures (33, 35, 37, 38, 39, 41, and 43°C). Linear regression analysis of these data across temperature indicated that at 72 h of proliferation C/EBP*β* expression increased (*P *<* *0.001) as temperature increased in both p. major (slope: 0.19) and b. femoris (slope: 0.02) satellite cells. However, the linear response of p. major satellite cells was significantly greater (*P *<* *0.001) across temperature compared to b. femoris cells. At 48 h of differentiation, the expression of C/EBP*β* in p. major satellite increased (*P *<* *0.001) with increasing temperature (slope: 0.06). In contrast, C/EBP*β* expression in b. femoris satellite cells linearly decreased (slope: −0.05; *P *<* *0.001) with increasing temperature at 48 h of differentiation.

At 72 h of proliferation, the expression of PPAR*γ* was significantly higher (*P *<* *0.001) in p. major satellite cells at 33 and 35°C compared to b. femoris cells, while b. femoris satellite cells had greater (*P *≤* *0.01) PPAR*γ* expression at 38, 39, and 43°C (Fig. 4C) compared to p. major satellite cells. Expression of PPAR*γ* at 72 h of proliferation decreased (*P *<* *0.001) as temperature increased (slope: −0.19) in p. major satellite cells. In contrast, PPAR*γ* expression at 72 h of proliferation in b. femoris cells did not (*P *=* *0.63) have any significant linear trend (slope: 0.003) across temperature (Fig. 4C). At 48 h of differentiation, p. major cells consistently expressed higher (*P *≤* *0.004) levels of PPAR*γ* than b. femoris cells (Fig. 4D) at all temperatures. The expression of PPAR*γ* increased linearly (slope: 0.04) in p. major cells at 48 h of differentiation with temperature (*P *≤* *0.02), while the expression in b. femoris cells linearly decreased (slope: −0.19) with temperature (*P *<* *0.001). Expression of the downstream adipogenic gene C/EBP*α* was assayed, but was not expressed at biologically significant levels (data not shown).

### Effect of temperature on apoptosis

The percentage of early and late apoptotic satellite cells was measured at 48 h of proliferation, and at 24 and 48 h of differentiation in p. major and b. femoris satellite cells at temperatures below and above 38°C. Both early apoptotic cells (Annexin-V-PE^+^/7-AAD^−^) and late apoptotic cells (Annexin-V-PE^+^/7-AAD^+^) at all temperatures and for both satellite cell types were less than 1.0% which was not biologically significant. However, a few statistically significant differences were observed. There were fewer (*P *≤* *0.03) b. femoris satellite cells incubated at 33°C in early stages of apoptosis compared to the control at 24 and 48 h of differentiation (Table 4). The percentage of cells in late apoptosis in p. major and b. femoris cells grown at 38 or 33°C was not significantly different (*P *≥* *0.05) across time in any culture conditions. Satellite cell type and temperature also did not affect apoptosis at 48 h of proliferation or 24 h of differentiation. However, at 48 h of differentiation there was a small percentage of p. major satellite cells incubated at 38°C in the late phase of apoptosis and this resulted in a significantly higher (*P *≤* *0.008) percentage of apoptotic cells compared to other experimental conditions that had no measurable apoptosis.

**Table 4:** Effect of temperature on apoptosis at 38 and 33°C during proliferation (P) and differentiation (D)*

Culture time	PM 38°C	PM 33°C	BF 38°C	BF 33°C
Early apoptosis†				
48hP	0.28 ± 0.06^a^,^x^	0.19 ± 0.03^a^,^x^	0.16 ± 0.04^a^,^y^	0.20 ± 0.04^a^,^x^
24hD	0.25 ± 0.07^ab^,^x^	0.26 ± 0.03^ab^,^x^	0.35 ± 0.03^a^,^x^	0.18 ± 0.03^b^,^x^
48hD	0.25 ± 0.04^ab^,^x^	0.19 ± 0.03^b^,^x^	0.31 ± 0.06^a^,^x^	0.18 ± 0.04^b^,^x^
Late apoptosis‡				
48hP	0.04 ± 0.02^a^,^x^	0.03 ± 0.02^a^,^x^	0.01 ± 0.01^a^,^x^	0 ± 0^a^,^x^
24hD	0.06 ± 0.03^a^,^x^	0.03 ± 0.02^a^,^x^	0.04 ± 0.02^a^,^x^	0.01 ± 0.01^a^,^x^
48hD	0.04 ± 0.02^a^,^x^	0 ± 0^b^,^x^	0 ± 0^b^,^x^	0 ± 0^b^,^x^

PM, pectoralis major; BF, biceps femoris

a-c Means across cell type and temperature at each sampling time; values without common letters are different (*P *<* *0.05).

x-y Means across sampling times for each cell type and temperature combination; values without common letters are different (*P *<* *0.05). Early and late data were analyzed separately.

* Apoptosis was quantified through flow cytometry readings of Annexin-V conjugated to phycoerythrin (Annexin-V-PE) and 7-amino-actinomycin (7-AAD) labeling.

† Early apoptosis, mean percent of cell population (±SEM) in early apoptosis (Annexin-V-PE^+^/7-AAD^−^).

‡ Late apoptosis, mean percent of cell population (±SEM) in late apoptosis (Annexin-V-PE^+^/7-AAD^+^).

At the highest temperature of 43°C, the percentage of p. major cells in early apoptosis was elevated (*P *≤* *0.04) at 43°C compared to the p. major cells incubated at 38°C at both 48 h of proliferation and 24 h of differentiation (Table 5). Across time, the percentage of early apoptotic p. major satellite cells incubated at 38°C was not different **(***P *>* *0.28**)** from 48 h of proliferation to 24 h of differentiation. However, from 24 h of differentiation to 48 h of differentiation the percentage of early apoptotic cells increased (*P *≤* *0.005). An increase in the percentage of early apoptotic cells was also detected at 48 h of differentiation in the 38°C b. femoris (*P *≤* *0.04) satellite cells. Comparing across cell type, the percentage of p. major late apoptotic cells incubated at 43°C was slightly elevated (*P *≤* *0.05) compared with the p. major satellite cells incubated at 38°C at 24 h of differentiation. Across time, the percentage of p. major late apoptotic cells incubated at 38°C increased (*P *≤* *0.02) slightly at 48 h of differentiation compared to both 48 h of proliferation and 24 h of differentiation. Apoptosis results for the p. major and b. femoris satellite cells incubated at 35, 37, 39, and 41°C at 48 h of proliferation and 24 and 48 h of differentiation were consistent with data at 33 and 43°C (data not shown).

**Table 5:** Effect of temperature on apoptosis at 38 and 43°C during proliferation (P) and differentiation (D)*

Culture time	PM 38°C	PM 43°C	BF 38°C	BF 43°C
Early apoptosis†				
48 hP	0.31 ± 0.05^b^,^y^	0.51 ± 0.07^a^,^x^	0.36 ± 0.06^ab^,^y^	0.46 ± 0.08^ab^,^x^
24 hD	0.28 ± 0.03^b^,^y^	0.63 ± 0.07^a^,^x^	0.50 ± 0.06^a^,^xy^	0.59 ± 0.08^a^,^x^
48 hD	0.61 ± 0.13^a^,^x^	0.49 ± 0.09^a^,^x^	0.59 ± 0.10^a^,^x^	0.51 ± 0.09^a^,^x^
Late apoptosis‡				
48 hP	0.06 ± 0.02^a^,^y^	0.09 ± 0.02^a^,^x^	0.08 ± 0.03^a^,^x^	0.08 ± 0.02^a^,^x^
24 hD	0.06 ± 0.02^b^,^y^	0.14 ± 0.03^a^,^x^	0.05 ± 0.03^b^,^x^	0.08 ± 0.03^ab^,^x^
48 hD	0.14 ± 0.03^a^,^x^	0.14 ± 0.03^a^,^x^	0.10 ± 0.03^a^,^x^	0.13 ± 0.03^a^,^x^

PM, pectoralis major; BF, biceps femoris.

a-c Means across cell type and temperature at each sampling time; values without common letters are different (*P *< 0.05).

x-y Means across sampling times for each cell type and temperature combination; values without common letters are different (*P *< 0.05). Early and late data were analyzed separately.

* Apoptosis was quantified through flow cytometry readings of Annexin-V-conjugated to phycoerythrin (Annexin-V-PE) and 7-amino-actinomycin (7-AAD) labeling

† Early apoptosis, mean percent of cell population (±SEM) in early apoptosis (Annexin-V-PE^+^/7-AAD^−^).

‡ Late apoptosis, mean percent of cell population (±SEM) in late apoptosis (Annexin-V-PE^+^/7-AAD^+^).

## Discussion

Satellite cells are a multipotential heterogeneous population of stem cells that can be induced to follow cellular differentiation pathways other than muscle (Asakura et al. 2001; Shefer et al. 2004). Satellite cells from different muscle fiber types maintain intrinsic differences in cell culture and preferentially differentiate into the same fiber type from which they were isolated (Feldman and Stockdale 1991; Huang et al. 2006). Additionally, satellite cells from different muscle fiber types have different rates of proliferation and differentiation (McFarland et al. 1997; Collins et al. 2005; Manzano et al. 2011), as well as varying myogenic and adipogenic potential (Lagord et al. 1998; Siu et al. 2005; Yada et al. 2006; Powell et al. 2014a).

Due to the stem cell nature of myogenic satellite cells, they are capable of differentiating into alternate cellular lineages, including adipogenic and osteogenic cell types (Asakura et al. 2001; Shefer et al. 2004). Myogenic to adipogenic cell fate conversion may contribute to the increased fat deposition observed in poultry breast muscle (a muscle that does not normally contain adipocytes) with environmental stressors of nutrient restriction (Baziz et al. 1996; Powell et al. 2014a; Velleman et al. 2014) and temperature (Baziz et al. 1996; Lu et al. 2007; Piestun et al. 2011; Al-Musawi et al. 2012). Such conversions of satellite cells from a myogenic to adipogenic lineage may also contribute to increased intramuscular fat depots that negatively impact treatment in individuals with diabetes mellitus (Hilton et al. 2008; Gallagher et al. 2009; Vettor et al. 2009) and other metabolic or age-related diseases (Freda et al. 2008; Hilton et al. 2008; Vettor et al. 2009).

A small subpopulation of myogenic satellite cells isolated from rats has been shown to spontaneously transition from a myogenic to adipogenic lineage (Rossi et al. 2010). Additionally, a number of studies have shown ectopic expression of the adipogenic master regulator PPAR*γ*, alone or in conjunction with C/EBP*α* in murine (Hu et al. 1995) or porcine (Yu et al. 2006) myoblast cultures causes myoblasts to reduce expression of myogenic regulatory factors, which prevents the myoblasts from differentiating into myotubes. The same murine cells grown under adipogenic conditions with ectopic expression of PPAR*γ* and C/EBP*α* differentiate into adipocytes (Hu et al. 1995). In bovine, PPAR*γ* and C/EBP*β* expression have been shown to promote adipogenic differentiation of muscle cells as well (Choi et al. 2013). The current study demonstrated an increase in C/EBP*β* expression in both p. major and b. femoris satellite cells with increased temperature at 72 h of proliferation and an increase in p. major cells at 48 h of differentiation, suggesting conversion of the myogenic satellite cells to an adipogenic lineage as temperature increased. The results from the current study demonstrated that a temperature increase as little as one degree above the control increased lipid accumulation. This finding is supported by Al-Musawi et al. (2012) who showed increased intramuscular fat deposition in the gastrocnemius muscle of 18-day-old chicks incubated for 3 days at one degree above normal in ovo incubation temperature. Taken together, the results from the present study support the conversion of myogenic satellite cells to an adipogenic pathway due to thermal manipulation.

Previous studies have compared cultures of chicken p. major and b. femoris myogenic satellite cells when exposed to a variety of stimuli to identify intrinsic differences in these cells. McFarland et al. (1997) demonstrated that b. femoris satellite cells have a greater proliferation rate than p. major satellite cells in response to increased amounts of chicken serum. However, in low-serum media, p. major satellite cells differentiated more quickly into myofibers than b. femoris cells (McFarland et al. 1997). In response to nutritional restriction, chicken p. major and b. femoris satellite cells differentially express myogenic regulatory factors (Powell et al. 2014b) and p. major satellite cells had a higher adipogenic potential than b. femoris satellite cells (Powell et al. 2014a). Comparisons of the p. major and b. femoris satellite cells in the current study demonstrated that p. major satellite cells are much more sensitive to temperature change than b. femoris satellite cells. Satellite cells isolated from the p. major muscle accumulated less lipid when cultured at temperatures below the control (38°C) and more lipid when incubated at temperatures above 38°C compared to b. femoris cells and also had a greater increase in C/EBP*β* expression compared to b. femoris satellite cells. These results agree with the findings of Powell et al. (2014a) that p. major satellite cells had a higher adipogenic potential resulting in increased lipid accumulation and generally higher PPAR*γ* and C/EBP*α* expression compared to b. femoris satellite cells during nutrient restriction. Taken together, these studies suggest inherent differences between satellite cells dependent on the fiber type of origin and a greater sensitivity of p. major satellite cells to environmental and nutritional stressors than b. femoris satellite cells.

The increased adipogenic potential of p. major versus b. femoris satellite cells is likely directly associated with the fiber type of origin. The avian p. major muscle contains predominantly type IIB anaerobic fibers and primarily derives energy from glycolysis of intramuscular glucose, whereas the b. femoris muscle contains a mix of type I and type II fibers and relies on both anaerobic glycolysis and energy efficient aerobic oxidation (Westerblad et al. 2010). An initial step of aerobic oxidation of carbohydrates, amino acids, or fatty acids results in the production of acetyl-CoA from pyruvate (Barrera et al. 1972). In an oxidative aerobic environment, acetyl-CoA will enter the citric acid cycle and be fully oxidized to produce energy. When ample energy sources are present in an aerobic cell, a build-up of acetyl-CoA triggers feed-back mechanisms that inhibit the production of acetyl-CoA (LaNoue et al. 1972) and initiates a series of reactions that convert pyruvate to glucose for energy storage. Alternately, a build-up of acetyl-CoA when the energy needs of the muscle are low or anaerobic conditions predominate will result in a portion of the acetyl-CoA being shunted into fatty acid production, where much of it is converted to malonyl-CoA (Volpe and Vagelos 1973). Both acetyl-CoA and malonyl-CoA become carbon donors to produce fatty acid chains, and therefore lipids. Although oxygen supply is not a factor in cell culture, the anaerobic versus aerobic energy metabolism preferences of the p. major and b. femoris muscle respectively, may be intrinsic to satellite cells from these muscles. This would not be unusual, as other properties, such as preference for differentiating into the same fiber type of origin is intrinsic to satellite cells (Feldman and Stockdale 1991; Huang et al. 2006). While acetyl-CoA in aerobic b. femoris cells will be required for energy production through the citric acid cycle or lead to the production of glucose, a build-up of acetyl-CoA in the primarily anaerobic p. major satellite cells would lead to fatty acid production. It is likely that increased temperature will result in increased metabolic demands on the muscle leading to greater production of pyruvate and acetyl-CoA. This would lead to increased lipid accumulation in both p. major and b. femoris satellite cells at higher temperatures compared to the control temperature. As described above, acetyl-CoA would not be used as efficiently in the anaerobic p. major satellite cells, therefore more acetyl-CoA would be directed to lipid production than in the b. femoris satellite cells, resulting in increased lipid accumulation in the p. major satellite cells with increased temperature.

Increased lipid accumulation in the anaerobic p. major satellite cells compared to the aerobic b. femoris satellite cells may be further exacerbated by less efficient breakdown of existing lipid in anaerobic cell types. The ability to utilize oxygen has been directly correlated to the relative number of mitochondria in skeletal muscle (Wang et al. 1999, 2004), therefore aerobic muscle fibers have a greater mitochondrial content than anaerobic muscle fibers. Utilization of fatty acids as an energy source in skeletal muscle varies depending on muscle conditioning and exercise, but the mitochondria is the primary location of fatty acid breakdown through *β*-oxidation in skeletal muscle (Helge et al. 2007; Houten and Wanders 2010). Therefore, the anaerobic p. major satellite cells will have fewer mitochondria and be less efficient at fatty acid breakdown than the aerobic b. femoris satellite cells, contributing to greater lipid accumulation with increased temperature.

In addition to causing the conversion of myogenic satellite cells to an adipogenic lineage, environmental stressors have been shown to cause skeletal muscle damage by inducing apoptosis (Pophal et al. 2003; Nakashima et al. 2004; Nierobisz et al. 2009). While a small amount of apoptosis is normal in development (Sandri and Carraro 1999), it is also a cause of muscle degradation in muscle atrophy (Allen et al. 1997; Siu et al. 2005; Adhihetty et al. 2007; Marzetti et al. 2008) and degeneration (Tidball et al. 1995; Sandri et al. 2001). Restricting nutrient availability has been shown to increase apoptosis in the pectoralis thoracicus muscle (Pophal et al. 2003; Nierobisz et al. 2009), as well as in p. major and b. femoris satellite cells (Powell et al. 2014a). Interestingly, unlike nutrient restriction, temperature did not affect the apoptosis of the p. major or b. femoris satellite cells during proliferation or differentiation in a biologically significant manner. Taken together, these data demonstrate that satellite cell muscle type affects adipogenic potential of satellite cells but does not influence apoptosis with temperatures colder or hotter than normal conditions.

In summary, a number of factors, including environmental stressors affect skeletal muscle development (Mozdziak et al. 2002; Nakashima et al. 2004; Friar and Locke 2007; Piestun et al. 2009, 2011), as well as adipogenic potential (Baziz et al. 1996; Lu et al. 2007; Al-Musawi et al. 2012; Powell et al. 2014a; Velleman et al. 2014). The results from the present study are important in developing strategies to maximize satellite cell-mediated muscle growth and to limit the conversion of satellite cells to an adipogenic lineage during temperature changes.

## Conflict of Interest

None declared.
